# Dispersion of Phonon Surface Polaritons in ZnGeP_2_: Anisotropy and Temperature Impacts

**DOI:** 10.1186/s11671-016-1270-7

**Published:** 2016-02-09

**Authors:** K. V. Shportko, A. Otto, E. F. Venger

**Affiliations:** Department of Semiconductor Heterostructures, V.E. Lashkarev Institute for Semiconductor Physics of NAS of Ukraine, 45 Nauki av., Kyiv, 03028 Ukraine; Institute for Experimental Physics and Condensed Matter, Heinrich Heine University Duesseldorf, Kufuerstenstr. 32, Ellenz-Poltersdorf, 56 821 Duesseldorf, Germany

**Keywords:** Single crystal, Phonon, Surface polariton, Anisotropy, Low temperature

## Abstract

Zinc germanium diphosphide (ZnGeP_2_) is an attractive and promising functional material for different devices of the nano- and optoelectronics. In this paper, dispersion of phonon surface polaritons (PSPs) in ZnGeP_2_ has been studied in the 200–500-cm^−1^ spectral range at 4 and 300 K. Dispersion of “real” and “virtual” PSPs were calculated for *C*-axis being normal and parallel to the surface. Anisotropy in ZnGeP_2_ leads to the different numbers of PSP dispersion branches for different orientations of the sample. The temperature-dependent phonon contributions in the dielectric permittivity shift dispersion of the surface polaritons in ZnGeP_2_ to the higher wavenumbers at 4 K. We have shown that experimental dispersion of PSP is in agreement with theory.

## Background

In this study, we report on the impact of anisotropy and low temperature on the dispersion of surface polaritons in zinc germanium diphosphide (ZnGeP_2_), which is a promising material for the nano- and optoelectronics.

### Introduction

Among other ternary A^II^B^IV^C^V^_2_ compounds, ZnGeP_2_ stands out for its properties, such as great values of the coefficients of the nonlinear susceptibility and birefringence, which attract interest of researches.

Mechanical stability, resistance to moisture, aggressive environments, and good thermal conductivity characterize ZnGeP_2_ as an attractive and promising functional material for different devices of the nano- and optoelectronics. In [1], it has been discussed that diphosphides can be used in infrared converter systems. Experimental results of [2] prove that replacing CdTe by diphosphides can increase efficiency of the solar converters and simplify their mass deployment.

Therefore, the study of optical properties of ZnGeP_2_ is crucial for industrial applications of this material.

In the previous paper [3], we have reported on the lattice vibration behaviors of ZnGeP_2_ single crystals derived from the far-infrared (FIR) reflectance spectra in the temperature range of 4–300 K. From [3], one can notice that optical phonon behaviors in ZnGeP_2_ at low temperatures are similar to those in other diphosphides [4, 5]. These results show that surface polaritons in ZnGeP_2_ can be excited in the number of spectral ranges. However, for ZnGeP_2_, the surface polaritons practically have not been studied yet. Results of [6] have shown that temperature has an impact on the dispersion of surface phonon polaritons in binary diphosphide Zn_3_P_2_.

Here, we present the results of our study of the dispersion of the surface polaritons in ZnGeP_2_ at different temperatures.

## Methods

In the experiments, a set of samples of single crystals of ZnGeP_2_ cut into plates of size 2 × 4 × 0.5 mm was used. The samples of ZnGeP_2_ were oriented to (001) and (100) crystallographic planes. The orientation of the crystallographic planes was controlled by X-ray diffraction.

As an input data for the calculations of the dispersion of phonon surface polariton (PSP), we used reflectance data of ZnGeP_2_ from our study [3]. These spectra were measured at 300 K in the 200–500-cm^−1^ range, using a Bruker IFS 66v/s spectrometer with Hg lamp as the source of radiation with a resolution of 0.5 cm^−1^, 256 scans per 20 s employing polarized radiation were collected in each experiment. The temperature of sample varied in the 4–300-K temperature range. The angle of incidence of radiation was less than 10°. Spectra were measured at two orientations of the electrical vector *E* of the IR radiation with respect to the crystal: *E||c* and *E˩c*.

The method of attenuated total reflection (ATR) was firstly used for the excitation of surface waves in metals [7]. Since general principles of excitation of PSP by ATR have been described in [8], this method remains the easiest and the most reliable for excitation of surface polaritons.

We obtained the ATR spectra of PSP for ZnGeP_2_ in the 300–500-cm^−1^ frequency range using polarized radiation. To measure these data, we used FTIR spectrometer Bruker IFS 66 with accessory with grazing angle of the incidence equipped by Ge hemisphere as ATR element. The air gap between the investigated sample and the hemisphere varied from 1.5 to 2 μ.

## Results and Discussion

**Fig. 1:**
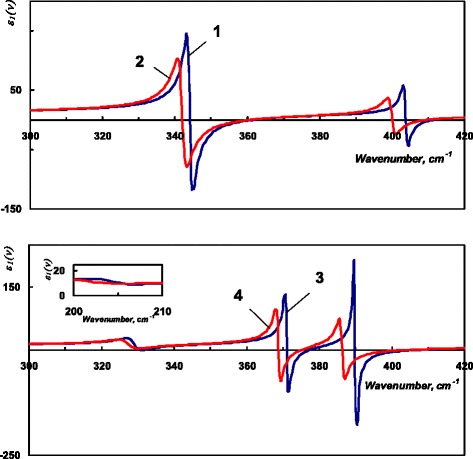
Real part of the dielectric function of ZnGeP_2_ single crystal. *Ε||c*: *1* 4 K, *2* 300 K; *Ε*˩*c*: *3* 4 K, *4* 300 K

In the tetragonal ZnGeP_2_, *B*_2_ (can be observed employing the radiation polarized parallel to the *c*-axis) and *E* (the radiation polarized normal to the *c*-axis) modes are infrared active. According to the data from [3], in the 200–500-cm^−1^ range, one can observe two *B*_2_ and four *E* modes. Spectral analysis of the reflectance data has been performed employing factorized model for the dielectric permittivity in the following form:

1 $$ \varepsilon \left(\nu \right)={\varepsilon}_{\infty }{\displaystyle \prod_{j=1}^n\left(\left({\nu}^2+i{\gamma}_{jl}\nu -{\nu}_{jl}^2\right)/\left({\nu}^2+i{\gamma}_{jt}\nu -{\nu}_{jt}^2\right)\right)}, $$

where *ε*_∞_ is high-frequency dielectric permittivity and *ν*_*jl*_ and γ_*jl*_ (*ν*_*jt*_ and γ_*jt*_) are the frequency and damping coefficient of the *j*th longitudinal (transversal) mode. Dispersion of the real part of the dielectric permittivity of ZnGeP_2_ obtained using model (1) at 4 and 300 K is presented in Fig. 1.

Results of spectral analysis of the reflectance data of ZnGeP_2_ at 4 and 300 K are shown in Table 1. Observed temperature shift of the phonon modes is in agreement with theoretical calculations from [9] and has been also observed in the reflectance spectra of Al_2_O_3_ [10] and Raman spectra of Si, Ge, and α-Sn [11].

**Table 1:** Input data for ZnGeP_2_

	*B*_2_ modes							
	4 K				300 K			
	*ε*_∞_		10.68		*ε*_∞_		10.04	
	*ν*_*L*_, cm^−1^	*γ*_*L*_, cm^−1^	*ν*_*T*_, cm^−1^	*γ*_*T*_, cm^−1^	*ν*_*L*_, cm^−1^	*γ*_*L*_, cm^−1^	*ν*_*T*_, cm^−1^	*γ*_*T*_, cm^−1^
1	362	2.0	343	1.9	361	2.1	340	3.5
2	412	2.2	403	1.1	409	2.8	400	1.8
	*E* modes							
	4 K				300 K			
	*ε*_∞_		10.05		*ε*_∞_		10.04	
	*ν*_*L*_, cm^−1^	*γ*_*L*_, cm^−1^	*ν*_*T*_, cm^−1^	*γ*_*T*_, cm^−1^	*ν*_*L*_, cm^−1^	*γ*_*L*_, cm^−1^	*ν*_*T*_, cm^−1^	*γ*_*T*_, cm^−1^
1	205	12.0	205	16.0	203	5.7	203	6.8
2	334	5.8	329	3.6	332	4.8	328	6.2
3	378	2.4	371	0.9	375	2.1	368	0.7
4	403	1.5	388	1.1	399	2.7	385	1.3

Before discussing the obtained results, we make a short review of influence of the anisotropy on the dispersion relation of PSP. The coordinate axes *x* and *y* lay in the sample’s plane, and *z* is normal to the surface. Let us denote the component of the dielectric tensor which is normal (parallel) to the optical axis of the crystal as *ε*_⊥_ (*ε*_||_). Neglecting the damping, we have analyzed this dielectric function applying conditions described in [8] and found spectral ranges of existence of the “real” (type 1) and “virtual” (type 2) PSP in ZnGeP_2_ at 4 and 300 K, which are shown in Table 2.

**Table 2:** Ranges of existence of PSP in ZnGeP_2_

	C*||*x	*C*||*y*	*C*||*z*
SPP 1		4 K	
	*ν* = 404 ÷ 406 cm^−1^	*ν* = 329 ÷ 332 cm^−1^	*ν* = 400 ÷ 403 cm^−1^
		*ν* = 369 ÷ 374 cm^−1^	
		*ν* = 386 ÷ 401 cm^−1^	
		300 K	
	*ν* = 400 ÷ 403 cm^−1^	*ν* = 327 ÷ 331 cm^−1^	*ν* = 404 ÷ 406 cm^−1^
		*ν* = 371 ÷ 376 cm^−1^	
		*ν* = 390 ÷ 404 cm^−1^	
SPP 2		4 K	
	*ν* = 344 ÷ 363 cm^−1^		*ν* = 201 ÷ 203 cm^−1^
	*ν* = 406 ÷ 409 cm^−1^		*ν* = 327 ÷ 331 cm^−1^
			*ν* = 369 ÷ 374 cm^−1^
			*ν* = 386 ÷ 399 cm^−1^
		300 K	
	*ν* = 342 ÷ 360 cm^−1^		*ν* = 204 ÷ 205 cm^−1^
	*ν* = 408 ÷ 412 cm^−1^		*ν* = 329 ÷ 333 cm^−1^
			*ν* = 371 ÷ 377 cm^−1^
			*ν* = 390 ÷ 403 cm^−1^

In the case of uniaxial media, according to the terminology of [6], “ordinary” PSP occurs when the optical axis *C* is parallel to the surface (*C*||*y*). The dispersion relation in this case (*ε*_*x*_ *= ε*_*z*_ *= ε*_⊥_) is as follows:

2 $$ {K}^2\kern1em =\kern1em {\varepsilon}_{\perp}\left(\nu \right)/\left({\varepsilon}_{\perp}\left(\nu \right)+1\right). $$

This dispersion relation involves only *ε*_⊥_, and it is of the same form as for PSP dispersion in isotropic media.

“Extraordinary” PSP occur in the two other configurations. The optical axis is perpendicular to the surface (*C*||*z*). The dispersion relation can be written down as follows:

3 $$ {K}^2\kern1em =\left({\varepsilon}_{\left|\right|}\left(\nu \right)\left(1-{\varepsilon}_{\perp}\left(\nu \right)\right)\right)/\left(\left(1-{\varepsilon}_{\perp}\left(\nu \right){\varepsilon}_{\left|\right|}\left(\nu \right)\right)\right), $$

where *ε*_*z*_ = *ε*_||_, *ε*_*x*_ = *ε*_⊥_. The optical axis is parallel to the surface of the sample and is parallel to the direction of propagating of PSP (*C*||*x*)

4 $$ {K}^2\kern1em =\left({\varepsilon}_{\perp}\left(\nu \right)\Big(1-{\varepsilon}_{\left|\right|}\left(\nu \right)\right)/\left(\left(1-{\varepsilon}_{\perp}\left(\nu \right){\varepsilon}_{\left|\right|}\left(\nu \right)\right)\right), $$

with *ε*_*x*_ = *ε*_||_, *ε*_z_ = *ε*_⊥_.

**Fig. 2:**
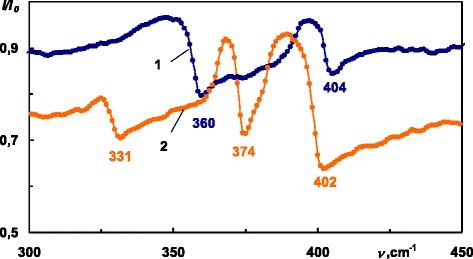
Experimental ATR spectra of ZnGeP_2_, 30°, 300 K. *1 C||x*, *2 C||y*

**Fig. 3:**
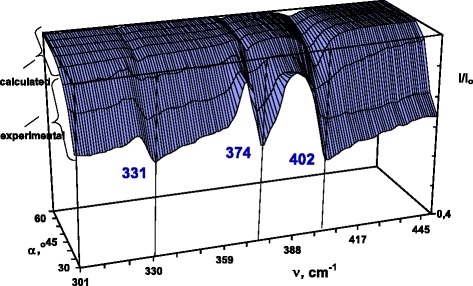
ATR surface *R*(*ν*,*α*) of Zn_3_P_2_, *C||y*

Experimental evaluation of the dispersion of surface polaritons has been performed applying the method of ATR, which was used for excitation of the surface waves in ZnGeP_2_ for *C*||*x* and *C*||*y* orientation of the sample at several angles of the incidence. Figure 2 presents experimental ATR spectra recorded at 30°. In the presence of the SP damping and dissipation of the electromagnetic wave energy, spectra show certain number of minima, whose wavenumbers correspond to the PSP. In Fig. 3, we show three experimental and five calculated ATR spectra (*C*||*y*) as the so-called ATR surface *R*(*ν*,*α*), which is a three-dimensional presentation of the system transmission that depends on the radiation frequency *ν* and angle *α*. This ATR surface *R*(*ν*,*α*) = *I*(*ν*,*α*)/*I*_0_(*ν*,*α*) has three “canyons” connected with a “pass.” *I*(*ν*, *α*) is the intensity of radiation passing through the “ATR element-gap-sample” system; *I*_0_(*ν*,*α*) is the intensity of the incident radiation onto the ATR unit. The depth of the “canyon” depends on the following system parameters: gap *d* between the ATR element and sample, radiation frequency *ν*, complex permittivity *ε*(*ν*,*k*) of the sample, and the refractive indexes of the ATR unit and gap. The surface polariton dispersion curves *ν*_*s*_(*k*) correspond to the “canyons”, i.e., to the set of ATR spectra minima. (Here, *ν*_*s*_ is the surface polariton frequency and *k* is the SP wave vector). Thus, we can distinguish three branches of PSP dispersion in ZnGeP_2_ within the measured spectral range. From the minima of ATR spectra, the PSP dispersion has been evaluated:

5 $$ k=\left(2\pi \nu /c\right)\;n \sin \alpha, $$

where *ν* is the frequency of the ATR spectrum minimum, *c* is the speed of light in a vacuum, and *n* is the refractive index of material of the ATR element.

**Fig. 4:**
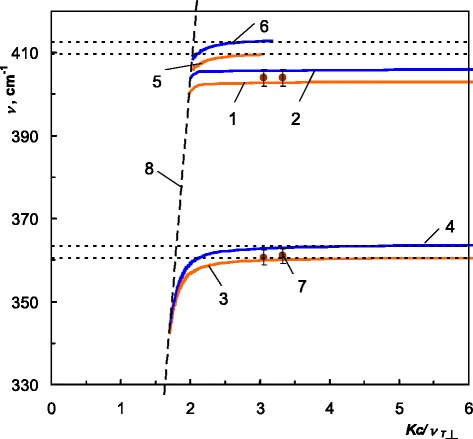
Dispersion of the PSP in ZnGeP_2_, *C||x*. “Real” PSP: *1* 300 K, *2* 4 K; “virtual” PSP: *3*, *5* 300 K, *4*, *6* 4 K; *7* experimental “real” PSP, 300 K, *8* “light line”

**Fig. 5:**
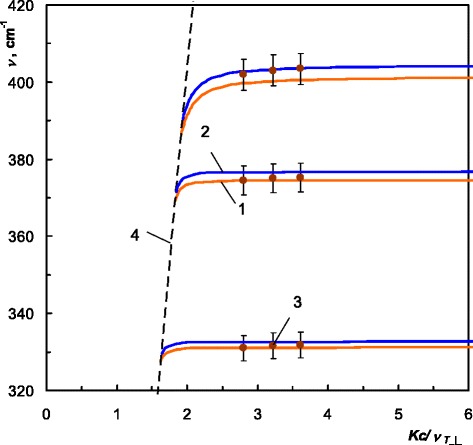
Dispersion of the PSP in ZnGeP_2_, *C||y. 1* “real” PSP, 300 K; *2* “real” PSP, 4 K; *3* experimental “real” PSP, 300 K; *4* “light line”

**Fig. 6:**
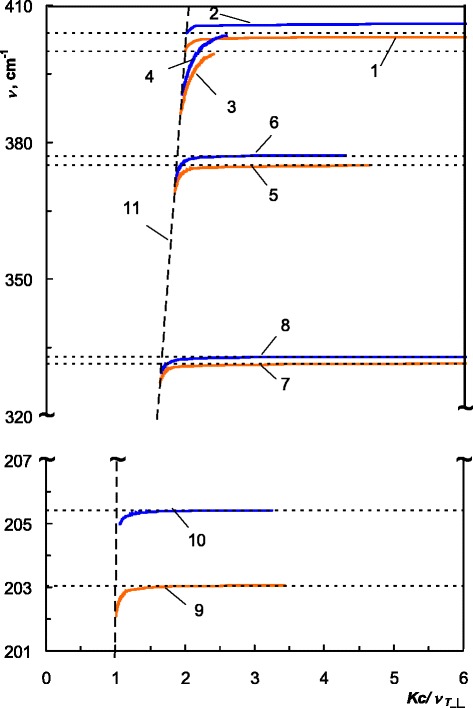
Dispersion of the PSP in ZnGeP_2_, *C||z*. “Real” PSP: *1* 300 K, *2* 4 K; “virtual” PSP: *3*, *5*, *7*, *9* 300 K, *4*, *6*, *8*, *10* 4 K, *11* “light line”

We have calculated dispersion of PSP in ZnGeP_2_ for three possible configurations applying Eqs. 2–4 in the spectral ranges shown in Table 2 at 4 and 300 K. We present the obtained PSP dispersion branches in Figs. 4, 5, and 6. To emphasize the influence of the temperature, we plot 4 K PSP dispersion curves in blue and 300 K in orange.

For configuration *C*||*x*, we obtained one branch of the dispersion of the “real” PSP and two branches of the dispersion of the “virtual” PSP. The “real” PSP branch goes to the infinity wave vector within the specified spectral range. The “virtual” dispersion branches terminate at the cross points of the polariton dispersion with the dispersions of the longitudinal optical phonons:

1. 412 cm^−1^, 4 K; 409 cm^−1^, 300 K;
2. 362 cm^−1^, 4 K; 361 cm^−1^, 300 K.

As already mentioned, for *C*||*y*, the dispersion relation is similar to the isotropic case, since only *ε*_⊥_ is involved in Eq. (2). Therefore, the quantity of dispersion branches is equal to the quantity of the Reststrahlen bands for *E˩c*. We obtained three dispersion branches of “real” PSP for *C*||*y* orientation.

We have obtained five PSP dispersion branches for *C*||*z* case: one branch of the dispersion of the “real” PSP and four branches of the dispersion of the “virtual” PSP, which terminate at the cross points of the polariton dispersion with the dispersions of the longitudinal optical phonons:

1. 203 cm^−1^, 4 K; 205 cm^−1^, 300 K;
2. 332 cm^−1^, 4 K; 334 cm^−1^, 300 K;
3. 375 cm^−1^, 4 K; 378 cm^−1^, 300 K;
4. 399 cm^−1^, 4 K; 403 cm^−1^, 300 K.

Due to the temperature shift of the phonon frequencies of ZnGeP_2_ to the higher wave numbers [3], almost all branches of the 4 K dispersion lay above the corresponding branches at 300 K. The temperature-dependent phonon contributions in the dielectric permittivity, which are described by frequency, damping, and strength in frames of Lorentz model [3], affect dispersion of the surface polaritons in ZnGeP_2_.

As one can notice in Figs. 4, 5, and 6, experimental PSP dispersion at 300 K calculated using Eq. (5) and shown in dots is a good agreement with the corresponding calculated dispersion branches. This agreement serves as a confirmation of 4 K PSP dispersion branches in ZnGeP_2_ which were calculated in the same manner.

## Conclusions

Thus, in this paper, dispersion of PSP in ZnGeP_2_ has been studied in the 200–500-cm^−1^ spectral range at 4 and 300 K. Dispersion of “real” and “virtual” PSP were calculated for *C*-axis being normal and parallel to the surface. Anisotropy in ZnGeP_2_ leads to the different numbers of PSP dispersion branches for different orientations of the sample. The temperature-dependent phonon contributions in the dielectric permittivity shift dispersion of the surface polaritons in ZnGeP_2_ to the higher wave numbers at 4 K. Experimental dispersion of PSP is in agreement with theory.
